# Imaging the Neuroimmune Dynamics Across Space and Time

**DOI:** 10.3389/fnins.2020.00903

**Published:** 2020-09-23

**Authors:** Micaël Carrier, Marie-Ève Robert, Fernando González Ibáñez, Michèle Desjardins, Marie-Ève Tremblay

**Affiliations:** ^1^Axe Neurosciences, Centre de Recherche du CHU de Québec, Université Laval, Québec City, QC, Canada; ^2^Axe Oncologie, Centre de Recherche du CHU de Québec, Université Laval, Québec City, QC, Canada; ^3^Department of Physics, Physical Engineering and Optics, Université Laval, Québec City, QC, Canada; ^4^Department of Molecular Medicine, Université Laval, Québec City, QC, Canada; ^5^Division of Medical Sciences, University of Victoria, Victoria, BC, Canada

**Keywords:** microscopy, microglia, fluorescence microscopy, electron microscopy, positron emission tomography, magnetic resonance imaging

## Abstract

The immune system is essential for maintaining homeostasis, as well as promoting growth and healing throughout the brain and body. Considering that immune cells respond rapidly to changes in their microenvironment, they are very difficult to study without affecting their structure and function. The advancement of non-invasive imaging methods greatly contributed to elucidating the physiological roles performed by immune cells in the brain across stages of the lifespan and contexts of health and disease. For instance, techniques like two-photon *in vivo* microscopy were pivotal for studying microglial functional dynamics in the healthy brain. Through these observations, their interactions with neurons, astrocytes, blood vessels and synapses were uncovered. High-resolution electron microscopy with immunostaining and 3D-reconstruction, as well as super-resolution fluorescence microscopy, provided complementary insights by revealing microglial interventions at synapses (phagocytosis, trogocytosis, synaptic stripping, etc.). In addition, serial block-face scanning electron microscopy has provided the first 3D reconstruction of a microglial cell at nanoscale resolution. This review will discuss the technical toolbox that currently allows to study microglia and other immune cells in the brain, as well as introduce emerging methods that were developed and could be used to increase the spatial and temporal resolution of neuroimmune imaging. A special attention will also be placed on positron emission tomography and the development of selective functional radiotracers for microglia and peripheral macrophages, considering their strong potential for research translation between animals and humans, notably when paired with other imaging modalities such as magnetic resonance imaging.

## Introduction

The immune system of the central nervous system (CNS) is essential to maintain its homeostasis [reviewed in Tay et al. (2017)]. Typically, this task is mainly performed by the resident immune cell population, microglia (Ginhoux et al., 2010; Tay et al., 2019). The role of microglia and other immune cells in the brain was uncovered using innovative methods allowing to image them *in situ* (Savage et al., 2019a) and *in vivo* (Davalos et al., 2005; Herz et al., 2012). Considering that immune cells like microglia are highly sensitive to changes in their microenvironment, non-invasive techniques that prevent their reaction during tissue preparation and imaging are necessary (Cătălin et al., 2017; Augusto-Oliveira et al., 2019). Immune cell reactivity can be assessed using morphological features (Savage et al., 2019a) or more sensitively by ultrastructure (El Hajj et al., 2019; Savage et al., 2019c). Overall, imaging techniques revealed that microglia play essential roles in the brain: as immune sentinels that clean toxic extracellular debris and repair damage to neighboring cells, but also as glial cells regulating neuronal and astrocytic/oligodendrocytic cell survival, differentiation, maturation, migration, as well as network formation and refinement with relation to neuronal activity (Ter Veer et al., 2017; Bordeleau et al., 2019; Tay et al., 2019). Once reactive, microglia play important roles in neuroinflammation –beneficial or detrimental depending on the context of disease or injury (Plemel et al., 2020). These roles of reactive microglia are exerted in cooperation with other glial and other innate immune cells (e.g., monocytes, neutrophils) as well as endothelial cells forming the blood-brain-barrier (BBB) (Sofroniew, 2015; Van Dyken and Lacoste, 2018; Bellver-Landete et al., 2019). Although monocytes were not shown to be present in the healthy adult mouse brain (Ginhoux et al., 2013; Prinz and Priller, 2014, 2017) they can be recruited rapidly in a wide panoply of contexts (D’Mello et al., 2009; Wohleb et al., 2015; Varvel et al., 2016; Niraula et al., 2018; Schneider et al., 2019). Once in the brain, monocytes tend to adopt a similar morphology as microglia and are often referred to as microglia-like cells even if they have a different origin, from the bone marrow while microglia come from the embryonic yolk sac (Leone et al., 2006; Varvel et al., 2012; Katsumoto et al., 2014). The adaptive immune system is also highly complex, with multiple cell types such as lymphocytes (T cells, B cells, and NK cells) playing a possibly important role in brain development, activity and plasticity notably through their secretion of neurotrophins (Kipnis et al., 2004; Lewitus and Schwartz, 2009; Morimoto and Nakajima, 2019). T cells in particular are often seen in the brain parenchyma in multiple sclerosis, both in human samples and its animal models of experimental autoimmune encephalomyelitis. In these autoimmune diseases, T cells are considered to change their behavior in a destructive way that causes demyelination and inflammation (Lehmann, 1998). Here, we will review the main imaging techniques that are currently available to study brain immunity, focusing on microglia as they are mainly studied, but also on monocytes and T cells, while providing examples of their complementary applications, and discuss the power of their combination into integrative studies.

## Imaging the Homeostatic Brain Using Photons

Microscopy techniques (brightfield, fluorescence, confocal, multiphoton, etc.) are commonly used in neuroimmunology to analyze the density, distribution and morphology of different cell types, including microglia, and their interactions one with another (Savage et al., 2019a). The examined cells often require markers to be visualized. Immunostaining is widely used to label the cells of interest across various types of samples, from animal models to human *postmortem* CNS. For example, double immunostaining against IBA1 and TMEM119 can be used to distinguish microglia (double positive; derived from embryonic yolk sac) from infiltrating peripheral macrophages (expressing IBA1 but not TMEM119; from bone marrow) (González Ibanez et al., 2019). One should note that TMEM119 expression is not stable in early development (until P20 in mouse) and can change in disease context such as multiple sclerosis, highlighting the importance of using a combination of markers including Fcrls, siglec-h, sall1, P2RY12 and more recently HEXB to identify microglia (Butovsky et al., 2014; Buttgereit et al., 2016; Konishi et al., 2017; Zrzavy et al., 2017; Masuda et al., 2020). Fluorescence can also label immune cells directly, without immunostaining, when markers are biologically expressed in transgenic animals (Martell et al., 2017; Daigle et al., 2018). Fate mapping of immune cells origin and trajectory, from the embryonic yolk sac or bone marrow to the CNS, and then function in the CNS can then be performed (Gomez Perdiguero et al., 2015). Reporter mice are especially useful when they are crossed one with another. As an example, the CX3CR1^+/GFP^;Thy1H^+/YFP^ model (Feng et al., 2000; Jung et al., 2000) labels microglia and neurons including synapses in two different colors. It allows to study dynamic relationships between the two cell types, which is particularly relevant for studying microglia-synapse structural interactions (Tremblay et al., 2010). Furthermore, fluorescent dyes can be injected *in vivo*, while cells can be stained *ex vivo* and introduced back into animals, which can be transgenic, to allow for their discrimination (Helmchen and Denk, 2005). Many different mouse models, discussed in detail in a comprehensive review (Theret et al., 2019), were developed to study the function of resident microglia and macrophages within the CNS. Parallel to this, photonic microscopy greatly evolved, providing always improved images, bypassing the spatial resolution limit from 350 nm to around 65 nm with super-resolution microscopy (Klar et al., 2001; Evilsizor et al., 2015), which is especially useful for studying synaptic interactions. We will next explore the main microscopy techniques that are commonly used in neuroimmunology and present emerging ones that could provide new insights. Advantages and limitations are compared in Table 1.

**TABLE 1:** Comparison of light microscopy techniques based on their advantages and limitations.

Method	Tissue preparation	Spatial resolution	Depth	Main uses	*In vivo* imaging	Advantages	Limitations	References
								
Epifluorescence	-Fixation -*In vivo*: cranial window	350–500 nm	<200 μm	Rapid verifications (i.e. staining outcome, morphology, distribution)	Possible, but not typically used	-Easy use -Low cost -Rapid -Multi-channels -Optical sectioning not required	-Light diffraction limit -Out-of-focus background fluorescence detection	Yang and Yuste, 2017; Bayerl et al., 2019
Slide scanner microscopes (brightfield or fluorescent)	-Fixation -Slide- mounted	350–500 nm	<200 μm	-Cell distribution, density and morphology -Surface area -3D reconstructions (whole brain or serial sections)	No	-Easy use -High-speed scanning of large tissue sections -Multi-channel	-Light diffraction limit -Out-of-focus background fluorescence detection	Mikula et al., 2007; Chen et al., 2014; Yang and Yuste, 2017; Roetzer et al., 2019
Confocal	-Fixation -Tissue clearing	200–800 nm	<100 μm	-2D imaging of tissue sections -3D reconstructions (z stacks)	Possible, but not typically used	-Reduction of out of focus background fluorescence detection -Multi-channels	-Photobleaching -Slow speed: not suitable for following rapid changes in dynamic phenomena	Graf and Boppart, 2010; Pérez-Alvarez et al., 2013; Oreopoulos et al., 2014; Villaseñor and Collin, 2017
2-photon	-Fixation -*In vivo*: thinned-skull or cranial window -Chronic *in vivo*: canula insertion	400–900 nm	>300 μm >500 μm in vitro	-Live *in vivo* imaging -Time-lapse imaging -3D reconstructions	Yes	-Reduction of out of focus background fluorescence detection -Reduction of light scattering -Increased spatial and temporal resolution -Multi-channels	-Photobleaching (less than confocal microscopes) -Slightly decreased resolution due the use of larger wavelengths	Santi, 2011; Pérez-Alvarez et al., 2013; Oreopoulos et al., 2014
STED	-Fixation -*In vivo*: thinned-skull or cranial window -Chronic *in vivo*: canula insertion	65–100 nm	10–15 μm	-Synapse and dendritic spine dynamics	Yes	-Subcellular resolution -Multi-channels	-High cost and difficult equipment accessibility -Low tissue-penetration depth	Klar et al., 2001; Westphal et al., 2008; Berning et al., 2012; Chéreau et al., 2015
Light-sheet	-Fixation -Tissue clearing -Organotypic cultures	270–1000 nm	750 μm (live tissue) 2 mm (cleared tissue)	-Whole small organisms imaging (i.e. mouse embryos) or brain -Overview of cellular networks -3D reconstructions	No	-High-speed scanning of large tissue sections -Reduced photobleaching -High tissue-penetration depth	-Not suitable for large organisms such as adult mice (i.e. suitable for mice up to P14) -Depth limit established by autofluorescence -Limited number of channels (max. 2–3)	Dodt et al., 2007; Santi, 2011; Fiolka, 2019; Wang et al., 2019

### Slide Scanning

**FIGURE 1:**
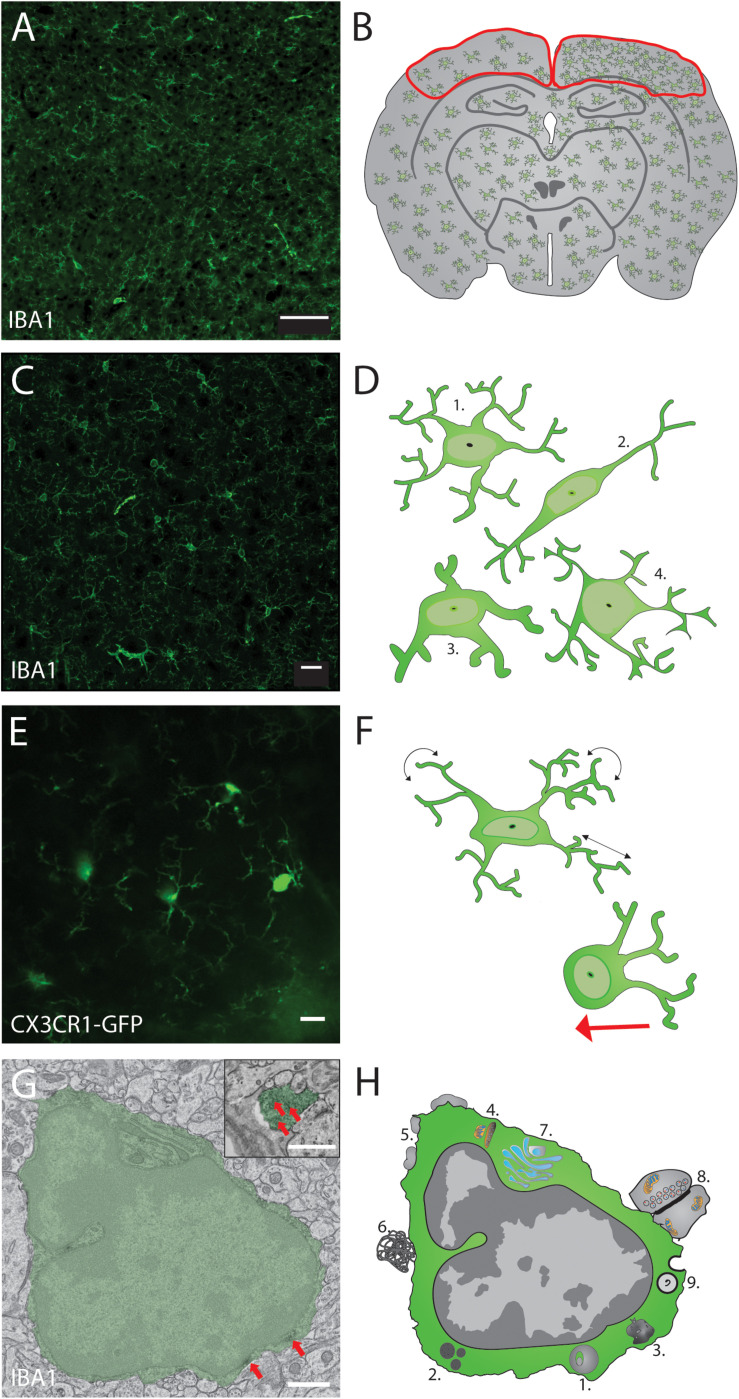
Proposed thorough structural analysis of the immune cells in the brain, exemplified with microglia. Optimal analysis of microglial density, distribution, morphology and ultrastructure using 4 different imaging modalities can allow to evaluate their cellular network, organellar function and health status. **(A)** Slide scanning can be performed to image microglial density and distribution throughout the brain (scale bar = 100 μm) while discriminating between different regions and layers of interest **(B)**. Microglial morphology can be assessed in **(C)** using confocal microscopy to measure quantitative changes in their cell body and arborization area (scale bar = 20 μm) to be then classified, as illustrated starting from the top in **(D)** into ramified, rod shape, hypertrophic and dystrophic phenotypes, an assessment not always feasible by slide scanner imaging due to the lower resolution and lack of 3D information. Microglial dynamics can be investigated using non-invasive two-photon *in vivo* imaging of CX3CR1+/GFP reporter mice (scale bar = 10 μm), as shown in **(E)** with a thinned-skull preparation allowing to assess microglial cell body mobility (red arrow in **F**) and process motility (black arrows) during normal physiological conditions in the intact mouse cerebral cortex. The ultrastructure of microglial cell body and processes can be analyzed using **(G)** transmission electron microscopy, as illustrated by a pseudocolored microglia (scale bar = 1 μm) that displays peroxidase staining in its cell body and processes against the IBA1 protein (red arrows). Cytologic events that can be quantified in microglia using different electron microscopy techniques are illustrated in **(H)** and include, notably, 1. Lysosomal inclusions, 2. Lipid bodies, 3. Lipofuscin granules, 4. Elongated or altered mitochondria, 5. Extracellular space pockets, 6. Extracellular digestion, 7. Endoplasmic reticulum/Golgi dilation, 8. Synaptic contacts, and 9. Phagolysosomal inclusions.

When answering questions about immune cell functions without knowing the specific CNS regions of interest involved, one can turn to high throughput imaging techniques such as slide scanning. This very convenient technique uses a mobile stage to automatically image regions of interest, from a series of CNS sections mounted on an entire microscopy glass slide, with a theoretical resolution of 900 nm (x-y). Whole mounts of mouse brain (Roetzer et al., 2019) and spinal cord (Bellver-Landete et al., 2019) were successfully imaged using this technique. It is ideal to perform quantitative analysis of microglial density and distribution, for instance, across large CNS tissue areas, as proposed within an experimental workflow in Figure 1A. One edge of the technique is the large amount of information provided in slide scanning images. Being formed of multiple tiles, these images allow the user to zoom in and out to visualize cellular density, distribution, morphology, intercellular relationships and other features of interest. The mosaic can be used to see the bigger picture of the cellular alterations or phenotypic changes as mentioned in Figure 1B. It can also be used to create new files containing smaller regions of interest with softwares such as QUPATH that could also be used for automatic analysis of density, distribution or morphology and intercellular relationships with machine learning (Bankhead et al., 2017). For instance, automatic analysis of slide scanning images was performed to characterize IBA1-immunopositive microglia/monocytes in traumatic brain injured mice at the site of injuries. The mosaic generated was used to count and classify these cells as “ramified” or “amoeboid” using machine learning, in less than 15 min compared to 5 h for a manual analyst. This faster process suggests strong potential for similar high-throughput analysis using human brain slices (Kyriazis et al., 2019). Slide scanning combined with machine learning is then a prime choice method for whole slices imaging in animal and human *postmortem* samples (Budde and Annese, 2013), considering that it removes the human bias when counting or tracing the contour of infiltrating monocytes or microglia from hundreds of slices.

### Confocal Microscopy

The biggest foe of fluorescent microscopy is light scattering, which limits its spatial resolution to about 300 nm (x-y) and 600 nm (z) at best. To circumscribe this problem, confocal microscopes increase resolution by only detecting the fluorescence emitted by the sample at the focal point. This feat is achieved by using a pinhole to reject light originating from outside the focal plane (Elliott, 2020). In neuroimmunology, confocal microscopy is commonly used to image the morphology of immune cells in three dimensions (3D), by acquiring z-stacks as presented in Figure 1C. Z-stacks are the key functionality of confocal microscopy. Allowing the 3D analysis of immune cells can give deeper insight when characterizing the shape of microglia, monocytes and T cells (Perego et al., 2013; Mittal et al., 2019). In Alzheimer’s disease pathology, for instance, T cell morphology can be 3D rendered in relation to neurons, which allowed the identification of “emperipolesis,” a phenomenon by which the T cell is actually inside the neuron, which might suggest neuronal damage associated with the foreign cell entry (Lodygin et al., 2019). In injuries, the morphology of microglia can also be effectively assessed from 3D reconstruction where the ramification density and arborization area can be analyzed for microglia close to injury site *versus* located in the periphery (Otxoa-de-Amezaga et al., 2019). Such changes in morphology, associated with microglial surveillance capacity and interactions with other parenchymal elements, including synapses, can be missed in typical epifluorescence images as smaller ramifications are difficult to discern with the reduced resolution. Thus, confocal microscopy is an effective tool to reveal the morphology of microglia and other cell types in 3D, as required to identify phenotypic changes, cellular alterations and functions, as illustrated in Figure 1D. In the case of microglia, their morphology is considered a direct indicator of their activity. For instance, amoeboid microglia tend to migrate in the brain while hypertrophic microglia tend to perform more phagocytosis than surveillant ones (Savage et al., 2019a; Tay et al., 2019). Using confocal microscopy, researchers can analyze various morphological changes like the formation of phagocytic cups at the end of microglia/monocyte processes, associated with the removal of engulfed newly proliferated cells and responsible for the development of juvenile play in adolescent rat (Sierra et al., 2010; VanRyzin et al., 2019). However, confocal microscopy is not often used in *in vivo* applications because of its limited penetration depth, which depends on the usable excitation wavelength (Graf and Boppart, 2010).

### Stimulated Emission Depletion (STED)

Microscopy increases image resolution by overcoming the light diffraction limit. Similar to other commonly used fluorescence microscopy approaches, STED involves the detection of fluorescence by point-scanning. A doughnut-like shape beam is used to deplete surrounding fluorophores in order to only read the fluorescence of the fluorophore of interest (Hell and Wichmann, 1994; Tardif et al., 2019) STED microscopy’s nanoscale resolution (65 nm in x-y and 150 nm in z) resulted in multiple applications, including the structural imaging of microglial and synaptic dynamics in fixed and live thick brain slices (Hein et al., 2008; Chéreau et al., 2015). Recent progresses further allowed to generate the first STED able to image efficiently *in vivo*, by adding components of a two-photon system. The use of a pulsed laser source provided better penetration, combined with the high spatial resolution of STED microscopy. With this approach, a greater number of small dendritic spines was imaged than with conventional two-photon microscopy, thus allowing to show a higher turnover of dendritic spines than previously envisioned (Pfeiffer et al., 2018). Other high-resolution microscopy techniques have been developed such as structured illumination microscopy (SIM) that uses a spatial modulation pattern to illuminate the sample, in which a software demodulation and filtering allows to overcome the diffraction limit with a factor two (Schermelleh et al., 2008). Stochastic optical reconstruction microscopy (STORM), where only a portion of fluorophores are excited on each cycle of laser emission, gives insight into their precise location at the nanoscale (Rust et al., 2006).

### Light-Sheet

While super-resolution techniques revolutionized the use of fluorescence microscopy by increasing its spatial resolution, light-sheet microscopy is a technique that was developed for faster imaging. It allows to perform high speed scanning of large CNS tissue volumes. The system requires a fluorescence detection setup that differs slightly from epifluorescence microscopy, since detection is performed transverse to the illumination by a thin sheet of laser light (Keller and Ahrens, 2015). This non-destructive imaging technique prevents sectioning interpretation artifacts. These artifacts arise when cells are cut in cross-section by vibratome, freezing microtome or cryostat. In addition, it offers the possibility to perform molecular analyses after imaging the fixed tissue (Glaser et al., 2017). Light-sheet fluorescence microscopy achieves a resolution of ∼26 μm (x-y) (Colombelli and Lorenzo, 2014) with 3D optical sectioning (up to ∼300 nm in *z*) and high-speed of imaging (2.7 × 10^4^ μm^3^s^–1^) (Lu C.-H. et al., 2019) that limits the effects of photobleaching compared to confocal microscopy (Santi, 2011; Power and Huisken, 2017). Light-sheet microscopy was used to visualize the entire adult mouse brain in health (Qi et al., 2019) and Alzheimer’s disease pathology (Liebmann et al., 2016). This emerging technique showed the 3D-distribution of neurons, microglia, the vasculature and tau proteins, throughout the entire brain parenchyma, using IDISCO to clear the tissue (Qi et al., 2019). Further iterations of IDISCO such as FDISCO or SHIELD could be employed as well (Renier et al., 2014; Park et al., 2018). However, light-sheet imaging techniques with *in vivo* whole mouse brain imaging capacity were not yet proposed (Dodt et al., 2007; Susaki et al., 2014).

### Multiphoton Microscopy

Is currently the technique of choice in neuroimmunology for *in vivo* studies. This technique uses a femtosecond pulsed laser that produces a very high density of photons in a very short time allowing for 2 or 3 photons, respectively, to play the same excitation role as a single photon of full energy. These 2 or 3 photons have half or one third of the quantum energy of a single photon since their wavelength is about two or three times longer than a single excitation photon (typically 800–950 nm for two-photon and 1100–1300 nm for three-photon microscopy) (Svoboda and Yasuda, 2006). Because longer wavelengths are more penetrating, one can explore phenomena deeper into biological tissues (Wang et al., 2018). The addition of energy from several photons takes place at the focal plane of the objective where a large number of photons is needed to make emerge this rare phenomenon, in a single point instead of a large double cone-like structure for single photon excitation. Therefore, the multiphoton technique preserves tissue integrity outside of the focal plane. With two-photon microscopy, the single focal point achieves a spatial resolution approximating 0.42 μm (x-y) and 0.81 μm (z) according to the numerical aperture of the objective (Soeller and Cannell, 1996; Diaspro et al., 2005; Zheng et al., 2019).

Seminal findings that dramatically changed the field of neuroimmunology were obtained with two-photon *in vivo* microscopy. A pioneer experiment conducted with this technique, in healthy conditions *versus* after a focal lesion generated with a two-photon laser, debunked the previous paradigm. Microglia are not resting cells in the absence of injury. Instead, they are extremely dynamic, with their processes constantly surveying the entire brain parenchyma with a time course that reaches 5.5 h in adult mice (Davalos et al., 2005; Nimmerjahn et al., 2005). In this seminal work conducted in anesthetized CX3CR1^+/GFP^ reporter mice, time-lapse images of fluorescent microglia were obtained through the skull, using minimally invasive thinned-skull method (Marker et al., 2010; Parkhurst et al., 2013). This achievement is reproduced in Figure 1E. A growing technology for two-photon microscopy is the resonant scanner, which allows acquisition speed to reach 30 frames per second. This high imaging speed is particularly useful for studying the real time activity of neuronal and glial cell populations via *in vivo* calcium imaging (Grewe et al., 2011; Olmedillas del Moral et al., 2019; Verkhratsky et al., 2019). Other types of microscopy techniques providing time-lapse imaging with high temporal resolution are extensively reviewed here (Mondal, 2014; Ueda et al., 2020).

## Beating the Spatial Resolution Limit Using Electrons

Electron microscopy (EM) generates images by interrogating a dehydrated sample with a beam of electrons, which are transmitted (transmission electron microscopy; TEM) or bounced back (scanning electron microscopy; SEM) using specialized detectors. The resulting beam reveals the ultrastructure of the investigated object, e.g., structure of protein, organelle, subcellular compartment or cell. By uncovering these elements from the atomic to the millimeter (and even centimeter) level, ultrastructural techniques have provided important information in neuroscience and neuroimmunology (Genoud et al., 2006; Knott and Genoud, 2013; Savage et al., 2018, 2019a). EM has allowed to shed light on microglia-neuron communication, revealing that 94% of microglial processes directly contact synaptic elements in the healthy adolescent mouse visual cortex (Tremblay et al., 2010). These ultrastructural insights and others contributed to defining the role of microglia in developmental synaptic pruning (Tremblay et al., 2010; Paolicelli et al., 2011; Schafer et al., 2012). The quadripartite synapse model, in which microglia and astrocytes regulate together synaptic function and plasticity, also arose from ultrastructural observations (Bennett, 2007; Tremblay et al., 2011, 2014; Schafer et al., 2013; Sierra and Tremblay, 2014).

### TEM

Electron microscopy can reach the resolution at <50 pm (x-y) for TEM compared to 3 Å for cryo-EM (Earl et al., 2017). This allows TEM to provide fine details regarding immune cells morphology, from cell body to finest and most distal processes. As illustrated in Figures 1G,H, this nanoscale resolution allows to identify signs of ‘intracellular’ activity (e.g., organellar changes, phagosomal inclusions) and extracellular activity (e.g., extracellular digestion of cellular debris, intercellular contacts with other glial cells, neurons, and synapses). The quantification of events for each cell type studied is essential to assess the situation of the cell, whether it is in steady state or overwhelmed by phagocytic inclusions requiring digestion, accumulated misfolded proteins in the endoplasmic reticulum or Golgi apparatus, stressed mitochondria or making increased synaptic contacts as previously evaluated for microglia across a range of contexts, including Huntington’s disease pathology (Savage et al., 2020). Additionally, an increasing body of evidence supports the idea that the microglial population is composed of diverse subpopulations endowed with unique intrinsic properties that perform different functions, and display a high degree of spatial and temporal specialization (Stratoulias et al., 2019). Evidence of this microglial heterogeneity was notably provided with TEM. The dark microglia, identified at the ultrastructural level by their electron dense cytoplasm and other markers of cellular stress, as well as extensive interactions with synapses (Bisht et al., 2016b), are rare in healthy young adult mice, but increase in number up to 10-fold with chronic unpredictable or social defeat stress, aging and other pathological contexts, including Alzheimer’s disease pathology (St-Pierre et al., 2020), showing the importance of this approach for the *in situ* study of microglial functional diversity.

### SBF-SEM

3D-EM allows to reconstruct organelles, cytoskeletal elements, subcellular compartments, and cellular relationships, among different cell types including immune cells *in situ* (Bolasco et al., 2018). Blocks of CNS tissue can be imaged using serial block face SEM, in which the tissue is sequentially imaged at a resolution of ∼10 nm (x-y), cut (25–50 nm thick)(z) with an ultramicrotome mounted inside the SEM chamber, realigned and imaged, to automatically generate z-stacks of serial images (Denk and Horstmann, 2004; Briggman and Bock, 2012; Peddie and Collinson, 2014; Yamasaki et al., 2014). This allows SBF-SEM to reconstruct multiple immune cells, neurons and astrocytes at the nanoscale (Calì et al., 2019). This technique is much faster than the method used in pioneering studies: serial section TEM to image synapses (Fiala and Harris, 2001) or microglial processes interacting with synapses (Tremblay et al., 2010), by aligning and segmenting series of ultrathin sections cut manually and reconstructed semi-manually with Reconstruct (Fiala, 2005). This method allowed the accurate comparison of nucleus to cell body ratio and mitochondrial distribution among the different brain cells, including between individual microglial cells, giving insight into the functional status of microglia and other cells in their 3D microenvironment (Savage et al., 2018). Microglial mitochondria analysis is promising based on reports of mitochondria defects in multiple neurodegenerative disease such as Parkinson’s disease, Huntington’s disease, Alzheimer’s disease and amyotrophic lateral sclerosis (Chaturvedi and Flint Beal, 2013). This whole cell analysis was further used to discriminate monocyte-derived cells from microglia based on their distinctive ultrastructural features in the brain of experimental autoimmune encephalomyelitis mouse models of multiple sclerosis. This distinction between microglia and monocyte-derived cells is normally difficult to achieve in a 2D plane but was possible in 3D while showing the specific interaction of monocytes at the Ranvier node, where they could be initiating the demyelination (Yamasaki et al., 2014).

### FIB-SEM

3D-EM is also possible using focused ion beam scanning electron microscopy (FIB-SEM) that removes as little as 3 nm of tissue after each block face image acquisition to create z-stack of images. FIB-SEM produces a higher 3D resolution reaching 3–5 nm (x, y, and z) (Briggman and Bock, 2012), which is required to study the fine geometry of organelles, phagosomes and autophagosomes and cytoskeletal elements at the expense of having a reduced field of view and slower acquisition speed compared with SBF-SEM (Heymann et al., 2006; Knott and Genoud, 2013; Peddie and Collinson, 2014; Savage et al., 2019b). With this level of resolution, FIB-SEM imaging of immune cells allows to discern between cellular elements that are partially surrounded by a microglial process, for instance during synaptic stripping, from the ones that are fully engulfed and internalized (phagocytosed). Different steps of phagocytosis process could be identified, from partial to complete engulfment, given the observation of microglia nibbling small pieces of axon terminals (i.e., performing trogocytosis) in the postnatal mouse hippocampus (Weinhard et al., 2018).

### Correlative Light and Electron Microscopy (CLEM)

Has seen its usage escalating over the past years (Begemann and Galic, 2016). Combining the minimally invasive *in vivo* imaging capacity of two-photon imaging with the nanometer resolution of serial section TEM revealed microglial ability to interact actively with pre- and post-synaptic elements, while some microglial cells had synaptic elements inside their phagosomes under normal physiological conditions (Tremblay et al., 2010). In the context of studying Alzheimer’s disease pathology, brightfield microscopy combined with TEM also allowed to image microglial diversity around amyloid plaques (Bisht et al., 2016a; El Hajj et al., 2019). Figure 1 proposes a correlative approach that combines four different modalities of microscopy to paint the most insightful portrait of microglial diversity. CLEM is a most promising method for investigating in 2D and even 3D microglial relationships with synapses throughout life, from development (Schafer et al., 2012) to aging (Beckman et al., 2019). Synapses can be imaged using confocal microscopy and their ultrastructural relationships with microglia and other immune cells can be imaged using TEM or even SEM with array tomography. This workflow with array tomography allows the captured images to be stitched into larger 2D mosaic, thus providing a better view of their general organization within CNS regions and layers. These images can be correlated with the evaluation of cognitive decline by measuring the loss of synapses but also the integrity of their structure in the prefrontal cortex of human donors (Henstridge et al., 2018). Other important protocols such as the Nanobody-Assisted Tissue Immunostaining for Volumetric Electron microscopy (NATIVE) can provide enhanced staining using single-domain nanobodies with better penetration into the tissue for large scale 3D reconstruction over the entire mouse hippocampus (Fang et al., 2018), and would allow to correlate data from the nanoscale to the brain-region level.

## Clinical View on Neuroimmune Imaging

Studying the human brain offers a different set of challenges compared to studying animal models. Several non-invasive techniques developed between 1960 and 1980 (Nutma et al., 2019), especially, allow to have a look at immune system activity inside the human brain. Some of the techniques currently used are positron emission tomography (PET) (Harrison et al., 2014) and magnetic resonance imaging (MRI) (Arnò et al., 2014; Harrison et al., 2015; Rollins et al., 2018) to analyze the integrity of the brain parenchyma and BBB (Kenk et al., 2015; Montagne et al., 2016).

### PET

Allows to visualize microglial activity by measuring the dynamic coupling of the radiotracer [11C]PBR28 or [11C]PK11195 to the translocator protein (TSPO). This protein localized to microglial mitochondria can be used as a proxy of their neuroinflammatory and phagocytic activity (Sandiego et al., 2015; Sucksdorff et al., 2019). This makes PET imaging useful to study immune cells *in vivo* using these radiolabels, but it lacks in spatial resolution (Albrecht et al., 2016). While [11C]PBR28 or [11C]PK11195 tracers are widely used, a range of different microglial receptors and signaling proteins can be targeted via PET radiotracers (Tronel et al., 2017). This approach allowed to observe live changes in microglial activity (notably associated with phagocytosis and/or release of pro-inflammatory mediators) in the human brain after peripheral injection of lipopolysaccharide, a bacterial endotoxin, that was studied to determine how the adaptive immune response exerts a direct effect on behavior between health and disease (Sandiego et al., 2015). For PET imaging, the nature of the radiotracer targets and their low expression levels at steady-state generally limit their use to study reactive functions (e.g., neuroinflammation and exacerbated phagocytosis), and not microglial functions in normal homeostatic conditions (Owen et al., 2012). Using PET in schizophrenic patients with [18 F]-FEPPA, another ligand for TSPO, neuroinflammation was assessed in schizophrenia patients after antipsychotic treatment, revealing no difference, thus suggesting that neuroinflammation happens in earlier stages of the pathology (Kenk et al., 2015). Efforts to find and develop new tracers for the study of non-inflamed microglia would represent a step of major importance for the future investigation of the microglial subpopulations engaged in different functions across a variety of homeostatic and neuroinflammatory states (Beaino et al., 2017; Villa et al., 2018). Other innate immune cells such as monocytes have also been imaged using PET with the tracer [111Indium] oxyquinoline which enters leukocyte cell membrane and provides an effective visualization in the mouse and human blood (Kircher et al., 2008). More mouse PET investigations have shown T cells which can also be labeled by [111Indium] oxyquinoline in animal models (Gong et al., 2011). Further investigation of monocytes and T cells is required in human to translate the findings from animal research. Clinical investigation using high spatial resolution imaging typically uses MRI.

### MRI

Is an established non-invasive imaging technique that is used in clinical and preclinical research studies, with various animal models, making it an excellent tool for translational research (Desjardins et al., 2019). MRI with gadolinium has been used in rodents to study BBB integrity and its role in depression together with blood oxygenation dynamics. This is particularly interesting as the BBB is emerging as an active interface between the periphery and the brain that modulates neuroimmune interactions differently between health and disease conditions (Menard et al., 2017). Further than BBB investigation, MRI is a crucial tool that provides a high-resolution view of brain structures across the whole parenchyma in the same session (Ladd et al., 2018). While it is not able to directly image immune cells, MRI is used to image the whole human or animal brain during neuroinflammation, giving insight into immune cells effects on the disruption of the neurovascular unit, demyelination as well as gray and white matter volume reduction (Quarantelli, 2015; Albrecht et al., 2016). One MRI imaging method, diffusion MRI, is widely used to image white matter tracts. This is achieved by measuring water molecule arbitrary movements in the parenchyma to map the cellular architecture of the white matter (Le Bihan and Iima, 2015). This technique was used similarly to PET for the analysis of neuroinflammation in schizophrenia patients. By measuring free-water abnormities with the MRI machine, authors showed limited extracellular space indicating less inflammation in the parenchyma during late schizophrenia stages (Pasternak et al., 2015), which supports the previous evidence.

With its ability to image during the lifetime of the subject, whether animal or human, MRI can be used in experimental settings to investigate disease progression longitudinally, which can then be correlated with *postmortem* immunostaining for microglia and monocytes (Neuwelt et al., 2009), in addition to various disease hallmarks (Justicia et al., 2008; Jacobs et al., 2012; Tang et al., 2018; Yi et al., 2019). In a rat model of glioma, tumors were imaged during growth progression while studying the microglial/macrophagic changes visualized using ultrasmall superparamagnetic iron oxides (USPIOs). Microglia/macrophages store iron, a mineral necessary for oxidative metabolism, synaptic plasticity, myelination and neurotransmitter synthesis (Nnah and Wessling-Resnick, 2018). USPIOs is also internalized by microglia and macrophages, making the USPIOs-filled cells appearing as dark spots on MRI images. The observations were then correlated with confocal imaging of microglia/macrophages taking up Texas Red-labeled USPIOs in the tumor zone using high spatial resolution confocal imaging (Fleige et al., 2001). Ultimately, correlative [18F]DPA-714-PET and MRI-USPIOs together with confocal imaging allowed to investigate microglia/macrophage properties in an experimental autoimmune encephalomyelitis mouse model of multiple sclerosis. This work showed that microglia/macrophages upregulate TSPO and IBA1 in the demyelinating regions during acute phases, suggesting their dysfunction as a contributing factor to the inflammation seen in multiple sclerosis (Coda et al., 2020).

## Conclusion

### A Case for Correlative Microscopy and New Development

Research focusing on the neuroimmune component of the CNS is fundamental to provide further information on the properly functioning system with the goal of identifying and understanding the complex subcellular, cellular and intercellular processes that become compromised, dysregulated, or exacerbated in disease, whether the involved cells are stressed, degenerating or senescent. There is a tremendous need to pair clinical PET and MRI together with *postmortem* CLEM, notably using slide scanning, confocal, TEM or SEM together within an integrative investigation. CLEM can bring researchers closer to the goal of providing further insights into the mechanisms that govern brain immunity and its important consequences on neuronal circuits and behavior across life. This collaborative endeavor aims to maintain health, as well as prevent and treat cancer, but also neurodevelopmental, neuropsychiatric, neurological and neurodegenerative diseases, in which the innate and adaptive immune cells have been critically implicated (Sierra and Tremblay, 2014; Rangachari et al., 2017; Lecours et al., 2018; Bordeleau et al., 2019). By bringing together complementary imaging approaches CLEM can allow researchers to pair the observations in order to provide unprecedented insights into the mechanistic underpinnings of CNS development, function and plasticity. From slide scanning and confocal microscopy to more specialized tools like two-photon imaging and the different emerging types of 3D-EM modalities (Briggman and Bock, 2012; Peddie and Collinson, 2014), STED and light-sheet microscopy, which are now on the rise, will likely become common practice as the technology becomes more accessible.

The perspective of the field is now at a point where several new technologies are just waiting to be used in neuroimmunology. One example of new technology that could provide insights into the role of T cells, in particular, is 4D electron microscopy. This cutting-edge type of microscopy detects photon-induced near-field signals measured at T cell surfaces. This principle allowed the evaluation by electromagnetic measurement of T cell activation and to correlate this finding with the major compatibility binding complexes measured from the near-field signals at the cell surface. These results demonstrated structural changes that serve as biomarkers of T cell sensitivity to immune challenges (Lu Y. et al., 2019). Other techniques are still not offering their advantages in neuroimmunology yet, such as holographic microscopy which brings the resolution of electron microscopy to the order of the Armstrong. This would raise the imaging of phagosomal inclusions highlighted in Figure 1H to a new level, where could be determined the content of these vesicles. Multi-isotope imaging mass spectrometry would also bring many possibilities to the field. This imaging technique is able to image and quantify molecules in the samples, which presents great potential to identify new molecular targets in the neuroimmunology field (Arrojo E Drigo et al., 2019; Madan et al., 2019).

## Author Contributions

MC was responsible for the manuscript plan and management, writing of the correlative and clinical imaging sections while taking care of the overall revision and formatting of the manuscript, and creating the figure included in the manuscript. M-ÈR was responsible for writing the fluorescent imaging section and creating the table. FG was in charge of writing the electron microscopy section of the manuscript. MD and M-ÈT were in charge of revising the manuscript and providing theoretical and writing support to all authors while M-ÈT contributed significantly to the structure and ideas content of the manuscript.

## Conflict of Interest

The authors declare that the research was conducted in the absence of any commercial or financial relationships that could be construed as a potential conflict of interest.
